# Signal-to-Noise Ratio of Brillouin Grating Measurement with Micrometer-Resolution Optical Low Coherence Reflectometry

**DOI:** 10.3390/s20030936

**Published:** 2020-02-10

**Authors:** Kazumasa Takada, Shin-ichi Satoh, Akiya Kawakami

**Affiliations:** Division of Electronics and Informatics, Faculty of Science and Technology, Gunma University, 1-5-1 Tenjin, Kiryu, Gunma 376-8515, Japan; sato_4141@yahoo.co.jp (S.-i.S.); t191d021@gunma-u.ac.jp (A.K.)

**Keywords:** fiber optics, fiber sensing, optical interference, nonlinear optics, four-wave mixing, Brillouin dynamic grating, Fourier-transform spectroscopy

## Abstract

Signal-dependent speckle-like noise was the dominant noise in a Brillouin grating measurement with micrometer-resolution optical low coherence reflectometry (OLCR). The noise was produced by the interaction of a Stokes signal with beat noise caused by a leaked pump light via square-law detection. The resultant signal-to-noise ratio (SNR) was calculated and found to be proportional to the square root of the dynamic range (DR) defined by the ratio of the Stokes signal magnitude to the variance of the beat noise. The calculation showed that even when we achieved a DR of 20 dB on a logarithmic scale, the SNR value was only 7 on a linear scale and the detected signal tended to fluctuate over ±14% with respect to the mean level. We achieved an SNR of 24 by attenuating the pump light power entering the balanced mixer by 55 dB, and this success enabled us to measure the Brillouin spectrum distributions of mated fiber connectors and a 3-dB fused fiber coupler with a micrometer resolution as examples of OLCR diagnosis.

## 1. Introduction

The Brillouin-enhanced four-wave mixing induced by counter-propagating pump lights and one probe light produces a backward Stokes light in a waveguide under test [1], which is assumed to be the reflection of the probe light by an acoustic wave or a dynamic Brillouin grating excited in the waveguide by the two pump lights. Optical time-domain reflectometry (OTDR) has been used to detect the reflection distribution, or Brillouin grating distribution, while changing the frequency difference between the pump lights to obtain the Brillouin spectrum distribution in an optical fiber [2,3,4,5,6]. A micrometer-scale spatial resolution is indispensable for diagnosing miniaturized optical circuits and modules with the same four-wave mixing technique. Since the spatial resolution of the OTDR is determined by the temporal width of the employed optical pulses, we should launch a picosecond optical pulse into an optical waveguide under test and detect return pulses without deformation by using a high-speed optical detector. However, such an attempt to increase the spatial resolution often results in increasing the electrical noise level due to the ultra-wide detection bandwidth over 10 GHz, and thus degrading the signal-to-noise ratio (SNR). To our knowledge, therefore, there have been no reports on the Brillouin grating measurement at a micrometer-scale spatial resolution with the OTDR method. To overcome the degradation of the SNR, we have proposed Brillouin grating-based optical low coherence reflectometry (OLCR) [7], which detects Stokes light by utilizing its interference with local oscillator (LO) light at a detection bandwidth less than 100 kHz. Since the center frequency of a Brillouin spectrum changes with strain, OLCR is expected to provide us with useful information on the micrometer-scale strain distributions of optical waveguide devices, optical modules and three-dimensional objects such as biological tissue.

In our previous experiments [8], two counter-propagating pump lights were produced by dividing the output from a DFB laser diode with a bandwidth of 1 MHz, and their optical path lengths at the joint of the mated fiber connectors under test were several meters in length and were adjusted so that they were equal. The phase of the acoustic wave travelling along the fiber was considered to fluctuate at frequencies of approximately several Hertz due to perturbations applied to their optical paths, and the resultant Stokes light wave, which was assumed to be the reflection of the probe light by the acoustic wave, should be coherent with the probe light wave in the same way as a Fresnel reflection. This means that there were no serious optical effects inducing signal-dependent (or speckle-like) variations in the Brillouin grating measurement. Actually, however, the measured distribution changed randomly along the fiber in the same way as the Rayleigh backscattering measurement [9], and the fluctuations were much larger than the background electrical noise level. The speckle-like noise was so high that it took a few hours to obtain one smoothed Brillouin grating distribution after averaging individual distributions, which we acquired with repetitive measurements, and we could not continue the measurements at different frequencies between the pump light waves while maintaining the same measurement condition. That is, the noise prevented us from measuring the Brillouin spectrum distribution in mated fiber connectors.

This paper shows theoretically and experimentally that the speckle-like noise was generated by the interaction of the Stokes signal with the beat noise via the square-law detection employed in the OLCR system. To reduce the variations, we had no choice but to further reduce the beat noise, which was generated by the beat between the LO light and one of pump light waves entering the balanced mixer. In Section 2 we describe the experimental setup of the OLCR system incorporating dispersive Fourier spectroscopy (DFS), where we used a configuration consisting of a polarization controller and a polarization beam splitter to block the pump light from entering the balanced mixer. We succeeded in greatly reducing the leaked pump light power by incorporating a fiber-optic polarizer.

First, in Section 3, we theoretically derive the dependence of the Stokes signal magnitude on the mean powers of the LO, probe and two pump lights. We compare the results with the experimental data in Section 4 to confirm that the OLCR detected the desired Stokes signal, which was produced by Brillouin-enhanced four-wave mixing. Then we derive the variance of the beat noise produced between the LO light and a leaked pump light together with that of the shot noise and that of the beat noise produced between the LO light and a reflection from a device under test (DUT). Finally, we calculate the SNR of the Brillouin grating measurement, which is defined as the ratio of the mean level to the standard deviation of the fluctuated Stokes signal. The first half of Section 4 compares the measured data with the theoretical results we derived in Section 3 to confirm experimentally that the origin of the speckle-like noise resulted from the interaction of the Stokes signal with the beat noise. In the latter half, we report that the SNR of a Brillouin grating distribution that we obtained with one measurement increased to 24 even at a micrometer spatial resolution as a result of the beat noise reduction that we realized by incorporating the polarizer. This reduction enabled us to obtain the Brillouin spectrum distributions of the mated optical connectors and a 3-dB fused fiber coupler.

## 2. Experimental Procedure

### 2.1. Experimental Setup

**Figure 1:**
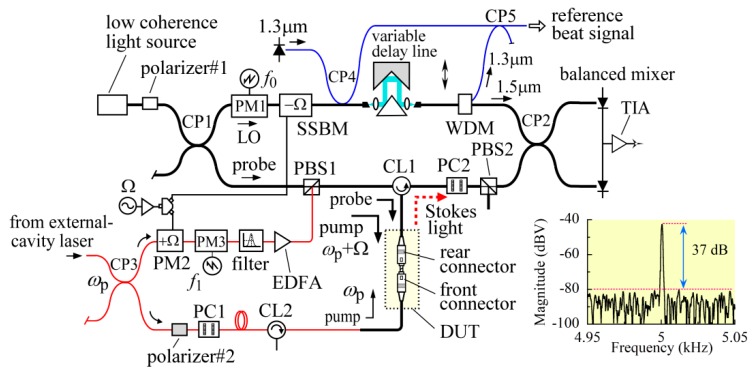
Schematic of Brillouin grating-based OLCR incorporating dispersive Fourier spectroscopy (DFS). The inset shows the spectrum of the Stokes signal acquired with an FFT signal analyzer. DUT: device under test, CL1 and CL2: optical circulators, CP1,CP2,, CP5: 3-dB fiber couplers, EDFA: erbium-doped fiber amplifier, PC1 and PC2: polarization controllers, PBS1 and PBS2: polarization beam splitters, SSBM: single-sideband modulator, PM1~PM3: phase modulators, WDM: wavelength-division multiplexer, LO: local oscillator, TIA: transimpedance amplifier, *ω*_p_: pump light frequency, Ω: down-conversion frequency, *f*_0_ = 145 kHz, *f*_1_ = 150 kHz.

The experimental setup we used was almost the same as the setup we reported in a previous paper [8]. To reduce the phase fluctuations of the induced acoustic wave as much as possible, we used an external-cavity laser as the pump light source, whose bandwidth was 100 kHz at 1550.12 nm. Since the laser output was only 1 mW, the output was amplified with an erbium-doped fiber amplifier, and the background spectrum of the amplified output was removed with a narrow-band filter. The Brillouin grating-based OLCR system consisted of the conventional OLCR setup for detecting the reflection from the mated fiber connectors as a DUT and an optical fiber loop for generating a Brillouin grating in the DUT by counter-propagating pump lights, as shown in Figure 1. 

The conventional OLCR part consisted of main and auxiliary interferometers. The former was a fiber-optic Mach-Zehnder interferometer with optical fiber couplers (CP1 and CP2), which was designed to detect the reflection whose optical frequency was down-converted by the acoustic wave induced in the DUT. We replaced the LiNbO_3_ intensity modulator that we employed earlier with a LiNbO_3_ phase modulator (PM3) to increase the signal level. We drove the phase modulators PM1 and PM3 at *f*_0_ = 145 kHz and *f*_1_ = 150 kHz, respectively, with sawtooth voltage waveforms so that the carrier frequency of the Stokes signal was *f*_c_ = *f*_1_ − *f*_0_ = 5 kHz. The photocurrent output from the balanced mixer was converted to a voltage with a transimpedance amplifier (TIA). The latter auxiliary interferometer was a fiber-optic Mach-Zehnder interferometer with optical fiber couplers (CP4 and CP5) and a 1 × 2 wavelength-division multiplexer (WDM), which shared a bulk-optic variable delay line with the former. We used a DFB laser diode operating at *λ*_0_ = 1316.077 nm as the light source, and the beat signal, which was produced during the translation of the linear stage installed in the variable delay line, enabled us to change the grid of the interferogram from equal time to equal path length increments.

In the optical fiber loop, the output from the pump light source was divided into two with an optical fiber coupler (CP3) for use as two counter-propagating pump lights. We describe the fixed frequency of the laser output as *ω*_p_ in the figure. To up-convert the frequency of the pump light by the same frequency Ω as the down-conversion frequency of the probe light, we applied phase modulation at Ω to the pump light with a LiNbO_3_ phase modulator (PM2) and extracted the up-converted light component with another narrow-band filter. The resultant pump light at *ω*_p_ + Ω was amplified with another erbium-doped fiber amplifier (EDFA), combined with the probe light at the polarization beam splitter PBS1, and launched into the DUT after passing through the optical circulator (CL1). The other laser output was launched into the DUT from the opposite direction for use as a pump light at *ω*_p_ after passing through the optical circulator (CL2). The optical path lengths of the counter-propagating pump light waves were adjusted so that they were equal at the joint between the mated fiber connectors.

We used a configuration consisting of a pair of polarization controllers (PC1 and PC2) and a polarization beam splitter (PBS2) to block the pump light at *ω*_p_ from entering the balanced mixer. We incorporated a fiber-optic polarizer (polarizer #2) in front of the PC1 to ensure that the pump light was linearly polarized with a negligibly small orthogonal component. By precisely adjusting the state of polarization (SOP) of the pump light with PC2, we were able to attenuate the pump light power to around 55 dB. While maintaining the attenuation and fixing the stage at a particular position, we detected the Stokes signal with an FFT signal analyzer and achieved a dynamic range of 37 dB as shown in the inset of Figure 1. The range was 14 dB greater than that in our previous paper, where the resolution and band shape of the analyzer were the same.

### 2.2. Dispersive Fourier Spectroscopy (DFS)

We introduced dispersive Fourier spectroscopy (DFS) [10,11] to numerically cancel the residual dispersion in the main interferometer and thus achieve the highest possible spatial resolution of 30 μm, which was dictated by the spectrum of the low coherence light output. We passed the balanced mixer output through an antialiasing filter with a cutoff frequency of 20 kHz. We set the controller to translate the linear stage at a speed of 400 μm/s and simultaneously oversampled the signal from the filter at a rate of 40k samples/s together with the reference signal from the auxiliary interferometer. The analytic signal of the Stokes signal was demodulated numerically by filtering the signal with a digital rectangular band-pass filter centered at 5 kHz and with a width of 630 Hz, digitally mixing the band-passed signal with in-phase and quadrature waveforms at 5 kHz, and low-pass filtering the result.

**Figure 2:**
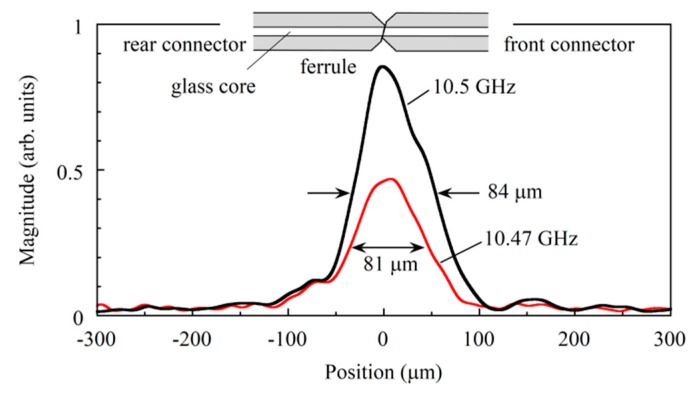
Brillouin grating distributions around the joint of the mated angle-polished fiber connectors, which were measured with the OLCR incorporating the DFS. The down-conversion frequencies applied to the SSBM were 10.47 and 10.5 GHz. The probe light and the pump light at *ω*_p_ + Ω propagated from rear to front connectors, whereas the pump light at *ω*_p_ propagated in the opposite direction.

In principle the absolute square of the analytic signal as a function of the time delay provided us with a Brillouin grating reflectogram. Before straightforwardly deriving the reflectogram from the demodulated signal, we changed the grid of the interferogram from equal time to equal path length increments, calculated the Fourier inverse transform, and removed the residual phase term of the transform, which was the origin of the degradation of the spatial resolution. We then calculated the Fourier transform to reconstitute the analytic signal after removing the noise that was distributed outside the range from 1527 to 1576 nm where the spectrum of the low coherence light was finite. Although the nominal spatial resolution of the constructed OLCR system was 30 μm, the best way to determine the actual spatial resolution was to measure the response of the OLCR system against a localized Brillouin grating whose length was assumed to be much shorter than the spatial resolution. However, it would be very difficult to generate such an ultimately narrow Brillouin grating artificially. Therefore, to check the spatial resolution beforehand, we measured the Stokes signal distribution (or Brillouin grating distribution) around the joint of angled physical contact fiber connectors that were mated with each other while changing the down-conversion frequency and found the narrowest Brillouin gratings whose full widths at half-maxima were 81 and 84 μm, at 10.47 and 10.5 GHz, respectively, as shown in Figure 2. The result shows that the actual spatial resolution was 81 μm or higher.

## 3. Calculations

**Figure 3:**
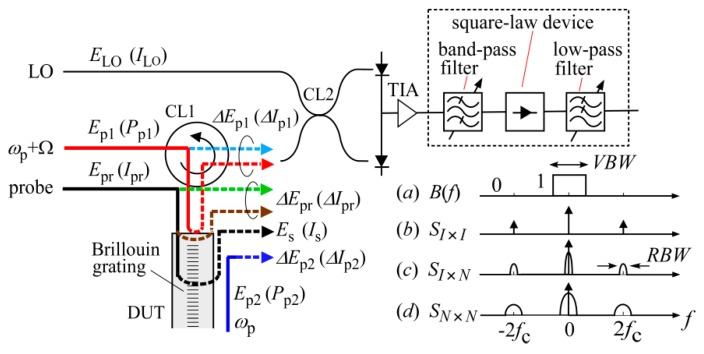
Propagation of the light waves Δ*E*_pr_, Δ*E*_p1_ and Δ*E*_p2_, which were generated from the probe light *E*_pr_, pump light *E*_p1_ at *ω*_p_ + Ω and *E*_p2_ at *ω*_p_, respectively, and entered the balanced mixer to produce beat noise. *I*_LO_ and *I*_pr_ are the mean photocurrents produced if the respective LO and probe lights would be detected with a photodiode. *P*_p1_ and *P*_p2_ are the light powers of the pump lights at *ω*_p_ + Ω and *ω*_p_, respectively. CL1: optical circulator, TIA: transimpedance amplifier. A schematic of the square-law detection and the relation between the constituent spectral densities are also shown. (*a*) shape of the low-pass filter whose width is *VBW*. The center frequency and the bandwidth of the band-pass filter are *f*_c_ and *RBW*, respectively. The constituent spectral density functions denoted as Equations (A1)–(A3) are represented by (*b*), (*c*) and (*d*), respectively.

We denote the electric fields of the LO, probe, pump light waves at *ω*_p_ + Ω and *ω*_p_, as *E*_LO_ + c.c., *E*_pr_ + c.c., *E*_p1_ + c.c. and *E*_p2_ + c.c., respectively, where c.c. denotes complex conjugate. In this paper *E*_LO_, *E*_pr_, *E*_p1_ and *E*_p2_ are referred to as the analytic signals of the respective electric fields. In Figure 3, the probe light *E*_pr_ passes through the circulator CL1, enters the DUT, and is reflected and down-converted by the Brillouin grating. The reflected light is referred to as the Stokes light that enters the balanced mixer after passing through the CL1, where the analytic signal is denoted as *E*_s_. In addition to the Stokes light, there are three kinds of light waves Δ*E*_pr_, Δ*E*_p1_ and Δ*E*_p2_, which enter the balanced mixer and generate beat noise. That is, due to the finite directivity of CL1, the probe light and the pump light at *ω*_p_ + Ω, are transmitted straight through it into the balanced mixer without entering the DUT, whereas they are Fresnel reflected at the endfaces of the DUT, return to CL1 and are combined with the respective transmitted light components. We denote the combined light wave that originates from the probe light as Δ*E*_pr_ and from the pump light as Δ*E*_p1_. Although the pump light at *ω*_p_ is attenuated by the polarization beam splitter PBS2, a small amount is transmitted through PBS2 and enters the balanced mixer. We denote the analytic signal of the pump light component entering the balanced mixer as Δ*E*_p2_.

Since the electric field *E*_s_ + Δ*E*_pr_ + Δ*E*_p1_ + Δ*E*_p2_ + c.c. is superimposed on that of the LO light by the 3-dB fiber coupler CP2, the instantaneous photocurrent from the balanced mixer is represented by:

(1) $$\begin{array}{l} I(t)=2i\alpha E_{\mathrm{LO}}(t)E_{S}{}^{*}(t)+2i\alpha E_{\mathrm{LO}}(t)\Delta E_{\mathrm{pr}}{}^{*}(t)\\ \;\;\;+2i\alpha E_{\mathrm{LO}}(t)\Delta E_{p1}{}^{*}(t)+2i\alpha E_{\mathrm{LO}}(t)\Delta E_{p2}{}^{*}(t)+c.c., \end{array}$$

where *i* = √−1 and *α* is a proportionality coefficient that converts the square value of the electric field to the photocurrent. The first term on the right-hand side of Equation (1) is the desired Stokes signal, whereas the second to fourth terms generate beat noise. Since *E*_LO_(*t*) is no longer correlated with Δ*E*_pr_(*t*) and Δ*E*_p1_(*t*) does not interfere with Δ*E*_p2_(*t*) because of their 10-GHz frequency difference, we assume the three kinds of noises to be statistically independent.

Since a Brillouin spectrum at a given location is obtained by measuring the mean square value of the Stokes signal as a function of the frequency difference between the pump lights, the voltage output from the TIA should be launched into the square-law detection system installed in the RF spectrum analyzer, which is shown enclosed by the dotted square in Figure 3. The detection system usually consists of a band-pass filter whose center frequency is set at the carrier frequency *f*_c_ = 5 kHz and whose bandwidth determines the variance of the beat noise, a square-law device to convert the signal to its squared value, and a low-pass filter to extract the slowly-varying component. Since the output of a conventional RF spectrum analyzer is scaled into a logarithmic magnitude in units of dBV or dBm, we converted the measured logarithmic values to the corresponding squared currents on a linear scale by using the gain of the TIA and compared them with the theoretical results. Hereafter, we calculate the magnitude of the Stokes signal and the power spectral density of the noise in units of A^2^ and A^2^/Hz, respectively.

### 3.1. Light Power Dependence of Stokes Signal

Since the coherent time of the broadband light from the OLCR source was much shorter than the response time of the detection system, the Stokes signal is obtained by performing statistical averaging over the first term on the right-hand side of Equation (1) as follows [12]:

(2) $$\begin{array}{l} I_{S}(t)=\frac{\alpha \omega_{1}\gamma_{e}\kappa_{1}\kappa_{2}}{2n^{2}\rho_{0}}\langle {\left|F(t)\right|}^{2}\rangle \exp\left\{i(\Delta \varphi +\omega_{1}\tau -\omega_{c}t)\right\}\\ \;\;\;\;\;\times \int_{-\infty }^{+\infty }r(\tau^{\prime })V^{*}(\tau -\tau^{\prime })\exp\left\{-i(\omega_{1}-\omega_{p})\tau^{\prime }\right\}d\tau^{\prime }+c.c., \end{array}$$

(3) $$V(\tau )=\int_{-\infty }^{+\infty }G\left(\omega_{1}+\omega \right)\exp\left(-i\omega \tau \right)d\omega /\int_{-\infty }^{+\infty }G\left(\omega_{1}+\omega \right)d\omega ,$$

when the delay induced by the variable delay line is *τ*. < > denotes the statistical average [13], *G*(*ω*_1_ + Ω) is the light spectrum of the broadband light whose center frequency is *ω*_1_, and *r*(*τ*) is the slowly-varying envelope of the acoustic wave. *γ_e_* and *ρ*_0_ are the electrostrictive constant and mean density of the waveguide material of the DUT, respectively. *n* is the refractive index of the waveguide, Δ*φ* is a constant and *ω*_c_ is the angular frequency, which is equal to 2π*f*_c_ where *f*_c_=5 kHz.

When deriving Equation (2) we assume that *F*(*t*) is the slowly-varying envelope of the electric field of the broadband light, and *κ*_1_ and *κ*_2_ are constants where the envelopes of the LO and probe lights are denoted as *κ*_1_*F*(*t*) and *κ*_2_*F*(*t*), respectively. Then the mean square values of the LO and probe lights are 2|*κ*_1_|^2^<|*F*(*t*)|^2^> and 2|*κ*_2_|^2^<|*F*(*t*)|^2^>, respectively, and the individual mean photocurrents, which would be measured if the individual lights were detected with a photodetector, are given by *I*_LO_ = 2|*α*|·|*κ*_1_|^2^<|*F*(*t*)|^2^> and *I*_pr_ = 2|*α*|*·*|*κ*_2_|^2^<|*F*(*t*)|^2^>. Similarly, *I*_p2_ denotes the photocurrent corresponding to the pump light at *ω*_p_.

*V*(*τ*) takes a finite value when *τ* is included in the range |*τ*|<*τ*_coh_, where *τ*_coh_ is the coherence time of the light source. When *r*(*τ*) changes with *τ* so slowly that *r*(*τ*ʹ) is considered to be a constant within the range of |*τ*-*τ*ʹ|<*τ*_coh_, Equation (2) can be simplified to:

(4) $$I_{S}(t)=\frac{\alpha \omega_{1}\gamma_{e}\kappa_{1}\kappa_{2}}{2n^{2}\rho_{0}\delta \nu_{\mathrm{LO}\times p}}\langle {\left|F(t)\right|}^{2}\rangle r(\tau )\exp\left\{i(\Delta \varphi +\omega_{p}\tau -\omega_{c}t)\right\}+c.c.,$$

where:

(5) $$\delta \nu_{\mathrm{LO}\times p}=\int_{0}^{+\infty }G(\nu )d\nu /G(\nu_{p})$$

is an effective width of the broadband light. Since the center of the band-pass filter is set at the carrier frequency of the Stokes signal, it can pass through the filter, and the output from the low-pass filter is denoted as:

(6) $$\langle I_{S}{}^{2}(t)\rangle =2{\left|\frac{\alpha \omega_{1}\gamma_{e}\kappa_{1}\kappa_{2}}{2n^{2}\rho_{0}\delta \nu_{\mathrm{LO}\times p}}\right|}^{2}{\langle {\left|F(t)\right|}^{2}\rangle }^{2}{\left|r(\tau )\right|}^{2}.$$

*V*(*τ*) is defined by Equation (3) by using the light spectrum *G*(*ω*_1_ + Ω), which is a function of the RF angular frequency *ω*. In the following subsection, we discuss the power spectral density of the beat noise as a function of the effective bandwidth. In Equation (5) we replace *G*(*ω*_1_ + Ω) with a new function *G*(*ν*), which is a function of the light frequency *ν*.

The slowly-varying envelope *r*(*τ*) of the acoustic wave in a steady state is [1]:

(7) $$r(\tau )=\frac{\varepsilon_{0}\gamma_{e}q^{2}Q_{1}A_{p1}A_{p2}^{*}}{\Omega_{B}^{2}-\Omega^{2}-i\Gamma_{B}\Omega },$$

where *ε*_0_ is the free-space permittivity, *q* is the wavenumber of the acoustic wave, Ω_B_ is the Brillouin frequency shift as a function of *τ*, and Γ_B_ is the Brillouin linewidth. We introduced a constant *Q*_1_ in Equation (7) to take account of the cross-sectional distributions of the acoustic and pump light waves in the waveguide of the DUT [14]. Since *A*_p1_ and *A*_p2_ are the envelopes of the pump light waves, the mean square values of the electric fields are 2|*A*_p1_|^2^ and 2|*A*_p2_|^2^, and therefore, the total mean powers that propagate in the DUT are *P*_p1_ = 2*Q*_2_|*A*_p1_|^2^ and *P*_p2_ = 2*Q*_2_|*A*_p2_|^2^, respectively, where *Q*_2_ is a constant. By substituting Equation (7) into Equation (6), we finally obtain:

(8) $$\langle I_{S}{}^{2}(t)\rangle =\frac{1}{32}{\left(\frac{\varepsilon_{0}\omega_{1}\gamma_{e}{}^{2}q^{2}Q_{1}}{n^{2}\rho_{0}\delta \nu_{\mathrm{LO}\times p}Q_{2}}\right)}^{2}\frac{I_{\mathrm{LO}}I_{\mathrm{pr}}P_{p1}P_{p2}}{{\left(\Omega_{B}^{2}-\Omega^{2}\right)}^{2}+{\left(\Gamma_{B}\Omega \right)}^{2}}.$$

The Stokes signal is proportional to the product of the mean powers of these four light waves. Since the spatial resolution increases with *δν*_LO×p_, Equation (8) means that the Stokes signal decreases approximately with the square of the resolution.

### 3.2. Current Noise Spectral Density

First, we derive the current noise spectral density of the second noise term Δ*I*(*t*) = 2*iαE*_LO_(*t*)Δ*E*_pr_^*^(*t*) + c.c. in Equation (1) by calculating its autocorrelation function. The whole calculation process is described in detail in [15]. By assuming that the electric fields obey a Gaussian random process [16], we can simplify their fourth-order moment as:

(9) $$\langle \Delta I(t)\Delta I(t+\tau )\rangle ={\left(2\alpha \right)}^{2}\int_{-\infty }^{+\infty }\exp\left(-2\pi if\tau \right)df\int_{0}^{+\infty }G_{\mathrm{LO}}(\nu )G_{\mathrm{pr}}(\nu +f)d\nu +c.c.$$

where *G*_LO_(*ν*) and *G*_pr_(*ν*) are light spectra of the LO and probe lights, respectively. Since *f* is a low frequency around the detection frequency at 5 kHz, and *ν* is the light frequency around 200 THz, we can make a good approximation of *G*_pr_(*ν* + *f*) ≈ *G*_pr_(*ν*) in Equation (9). Considering that the mean photocurrents of the LO and probe lights are *I*_LO_ = 2|*α*|<|*E*_LO_(*t*)|^2^> and Δ*I*_pr_ = 2|*α*|<|*E*_pr_(*t*)|^2^>, respectively, the current noise spectral density is obtained as:

(10) $$\sigma_{\mathrm{LO}\times \mathrm{pr}}^{2}=4I_{\mathrm{LO}}\Delta I_{\mathrm{pr}}\int_{0}^{+\infty }G_{\mathrm{LO}}(\nu )G_{\mathrm{pr}}(\nu )d\nu /\int_{0}^{+\infty }G_{\mathrm{LO}}(\nu )d\nu \int_{0}^{+\infty }G_{\mathrm{pr}}(\nu )d\nu$$

Since *G*_pr_(*ν*) has the same shape as *G*_LO_(*ν*) and shifts by only 10 GHz, which is much less than the light frequency *ν*, we can assume *G*_pr_(*ν*) to be proportional to *G*_LO_(*ν*) in Equation (10), and we obtain the final result of the spectral density for the beat between the LO and probe light as:

(11) $$\sigma_{\mathrm{LO}\times \mathrm{pr}}^{2}=4\frac{I_{\mathrm{LO}}\Delta I_{\mathrm{pr}}}{\delta \nu_{\mathrm{LO}\times \mathrm{pr}}},$$

where *δν*_LO×pr_ is another effective width of the broadband light defined by [17] as:

(12) $$\delta \nu_{\mathrm{LO}\times \mathrm{pr}}={\left(\int_{0}^{+\infty }G_{\mathrm{LO}}(\nu )d\nu \right)}^{2}/\int_{0}^{+\infty }G_{\mathrm{LO}}^{2}(\nu )d\nu .$$

The current noise spectral density of the third noise term 2*iαE*_LO_(*t*)Δ*E*_p1_^*^(*t*) + c.c. in Equation (1) is obtained by replacing Δ*I*_pr_ with Δ*I*_p1_ and *G*_pr_(*ν*) with *G*_p_(*ν*) in Equation (10), where Δ*I*_p1_ and *G*_p_(*ν*) are the mean photocurrent and spectrum of the pump light component Δ*E*_p1_. Since the origin of Δ*E*_p1_ is the coherent light from the laser source whose bandwidth is 100 kHz, *G*_p1_(*ν*) is approximated as a delta function *δ*(*ν*-*ν*_p1_) with *ν*_p1_ = (*ω*_p_ + Ω)/2π and the resultant width of Equation (12) becomes the effective width defined by Equation (5). Therefore, we obtain:

(13) $$\sigma_{\mathrm{LO}\times p1}^{2}=4\frac{I_{\mathrm{LO}}\Delta I_{p1}}{\delta \nu_{\mathrm{LO}\times p}}.$$

Similarly, the current noise spectral density of the fourth term in Equation (1) is:

(14) $$\sigma_{\mathrm{LO}\times p2}^{2}=4\frac{I_{\mathrm{LO}}\Delta I_{p2}}{\delta \nu_{\mathrm{LO}\times p}}$$

Then the total current noise spectral density including the shot noise effect is:

(15) $$\sigma^{2}=2e\left(I_{\mathrm{LO}}+\Delta I_{\mathrm{pr}}+\Delta I_{p1}+\Delta I_{p2}\right)+\sigma_{\mathrm{LO}\times \mathrm{pr}}^{2}+\sigma_{\mathrm{LO}\times p1}^{2}+\sigma_{\mathrm{LO}\times p2}^{2},$$

where *e* is the elementary charge. In Equation (15) we assume that the intensity noise of the broadband LO light is reduced to a negligible level by using balanced detection and the shot noise caused by the weak Stokes light is also negligible. It should be noted that the derived spectral density is the variance of the total noise observed when the bandwidth of the band-pass filter is unity. The last two terms in Equation (15) are characteristic of an OLCR system designed for Brillouin grating measurement.

The OLCR system was originally developed to measure the Rayleigh backscattering distribution in a silica-based waveguide. The Rayleigh backscatter signal was so weak that we defined the SNR of the Rayleigh backscattering measurement as the ratio of the mean level of the fluctuated Rayleigh backscatter signals to the current noise spectral density when estimating the minimum-detectable reflectivity at a unit detection bandwidth [18]. Instead, in this paper the ratio of the mean Stokes signal level to the variance is referred to as the dynamic range (DR):

(16) $$DR=\frac{\langle I_{S}{}^{2}(t)\rangle }{\sigma^{2}}$$

because the range would be observed on the display of an RF spectrum analyzer if the output of the TIA were connected to it and the spectrum around the carrier frequency of 5 kHz were measured, as shown by the inset of Figure 1.

### 3.3. Relationship between DR and SNR

The output from the band-pass filter is denoted as *I*_s_(*t*) + *N*(*t*), where *I*_s_(*t*) is a sine wave of the form *A*cos(2*πf*_c_*t* + *θ*) with a constant *A* as described by Equation (4), and *N*(*t*) is the fluctuating term of the total noise in Equation (1) whose variance is <*N*^2^(*t*)> = *σ*^2^. Then, the output from the square-law device is *Y*(*t*) = {*I*_s_(*t*) + *N*(*t*)}^2^ = *I*_s_^2^(*t*) + 2*I*_s_(*t*)*N*(*t*) + *N*^2^(*t*) whose slowly-varying component can pass the low-pass filter. The second term, 2*I*_s_(*t*)*N*(*t*) in *Y*(*t*), fluctuates greatly as the signal *I*_s_(*t*) increases, and this is the main origin of the speckle-like noise we observed with OLCR. The signal fluctuation is expressed by the standard deviation *σ_Y_* of the output from the low-pass filter, where the variance *σ_Y_*^2^ is written as the general form:

(17) $$\sigma_{Y}^{2}=\langle Y^{2}(t)\rangle -{\langle Y(t)\rangle }^{2}.$$

From the definition of *I*_s_(*t*) = *A*cos(2*πf*_c_*t* + *θ*), the signal from the low-pass filter output is <*I*_s_^2^(*t*)> = *A*^2^/2. Assuming that *I*_s_(*t*) and *N*(*t*) are statistically independent, we have <*I*_s_(*t*)*N*(*t*)> = 0, and so <*Y*(*t*)> in Equation (17) is written as:

(18) $$\langle Y(t)\rangle =\frac{A^{2}}{2}+\sigma^{2}.$$

Once the first term <*Y*^2^(*t*)> in Equation (17) is calculated for the low-pass filter output, it is certain that the SNR of the Stokes signal measurement is defined by:

(19) $$SNR=\frac{A^{2}/2}{\sigma_{Y}}.$$

The actual form of <*Y*^2^(*t*)> is derived in [19] where the band-pass filter has a Gaussian window. In our experiment, however, we used three types of windows to measure the magnitude of the signal and noise. One was a Gaussian window, which was installed in an analog RF spectrum analyzer, the second was the flat-top window of the FFT signal analyzer, which we used to measure the spectrum shown by the inset of Figure 1 and the current noise spectral density, and the third was the rectangular (or uniform) window that we employed in the DFS. Therefore, we calculated <*Y*^2^(*t*)> at an arbitrary window function *H*(*f*) according to the reference as shown in the Appendix A of this paper, and we found that:

(20) $$\langle Y^{2}(t)\rangle =\frac{A^{4}}{4}+2A^{2}\sigma^{2}+2\sigma^{4}$$

with any window function as long as the variance of the noise from the band-pass filter is *σ*^2^. By substituting equations (18) and (20) into Equation (17), we have:

(21) $$\sigma_{Y}^{2}=A^{2}\sigma^{2}+\sigma^{4}$$

and the SNR defined by Equation (19) is *SNR* = (*A*^2^/2)/√(*A*^2^*σ*^2^ + *σ*^4^). By substituting <*I*_s_^2^(*t*)> = *A*^2^/2 into Equation (16), we have *DR* = (*A*^2^/2)/*σ*^2^ and so the SNR is written with the DR as:

(22) $$SNR=\frac{DR}{\sqrt{2DR+1}}.$$

When *DR*>>1, as is usually the case, we have *SNR*≈√(*DR*/2) meaning that the SNR is proportional to the square root of the DR which increases in proportion to the product of the mean powers of the four light waves. On the other hand, the DR and thus the SNR degrade as the power of the pump light entering the balanced mixer increases, where the variance of the generated beat noise is proportional to the product of the mean photocurrents of the LO light and the leaked pump light. The square root dependence of the SNR on the DR means that even when we achieve a DR of 20 dB on a logarithmic scale, the SNR value is only 7 on a linear scale, which indicates that the detected signal tends to fluctuate over ±14% with respect to the mean level.

## 4. Experimental Results and Discussion

### 4.1. Power Dependence of the Stokes Signal

**Figure 4:**
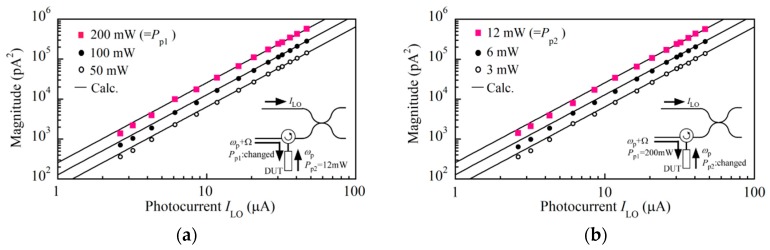
Magnitude of the Stokes signal as a function of the photocurrent *I*_LO_ of the probe light when (**a**) the power *P*_p1_ of the pump light at *ω*_p_ + Ω was 50, 100 and 200 mW, where the power *P*_p2_ of the pump light at *ω*_p_ was fixed at 12 mW, and (**b**) P_p2_ = 3, 6, and 12 mW, where *P*_p1_ was fixed at 200 mW. The solid lines were plotted by calculating *C*_s_*I*_LO_*I*_pr_*P*_p1_*P*_p2_ with *C*_s_ = 4.29 × 10^−5^.

We measured the Stokes signal at a distance of 2 cm from the joint of the mated fiber connectors with the FFT signal analyzer. We set the center frequency at 5 kHz and chose a flat-top window since there were small fluctuations in the carrier frequency caused by perturbations experienced by the main interferometer. The down-conversion frequency was 10.77 GHz. To measure the magnitude of the signal accurately, we averaged ten spectra, which we acquired with repetitive frequency scans and determined the peak in the mean spectrum by employing a least square fitting procedure. We converted the magnitude of the signal scaled in dBV units to pA^2^ units by using the gain of the TIA. The maximum powers of the pump lights at *ω*_p_ + Ω and *ω*_p_ were *P*_p1_ = 200 mW and *P*_p2_ = 12 mW, respectively. We increased the output power from the low coherence light source and measured the signal as a function of the photocurrent *I*_LO_ of the LO light as shown in Figure 4a,b. It is noted that when the power of the probe light was 114 mW, the photocurrent was *I*_LO_ = 47 μA. Figure 4a shows the measurement result at pump powers of *P*_p1_ = 50, 100 and 200 mW while *P*_p2_ was fixed at 12 mW, and Figure 4b shows the result at pump powers of *P*_p2_ = 3, 6 and 12 mW while *P*_p1_ was fixed at 200 mW, where logarithmic scales were used on both the horizontal and vertical axes. Since the variation of each data point was 10% with respect to the mean value, we did not add error bars to the log-log graphs. At each *I*_LO_ value, the signal increased twofold when the pump power of either *P*_p1_ or *P*_p2_ was doubled, and this dependence means that the signal was proportional to the product of the individual pump light powers, or *P*_p1_ × *P*_p2._

The signal dependence on *I*_LO_ for every pair of (*P*_p1_, *P*_p2_) agreed with the square function of *I*_LO_, which is shown by the solid line, and this dependence means that the signal was proportional to *I*_LO_^2^. As described in Section 3.1, the photocurrents of the LO and probe lights are defined by *I*_LO_ = 2|*α*|·|*κ*_1_|^2^<|*F*(*t*)|^2^> and *I*_pr_ = 2|*α*|*·*|*κ*_2_|^2^<|*F*(*t*)|^2^>, respectively. Since 2|*α*|<|*F*(*t*)|^2^> is the photocurrent corresponding to the output power from the low coherence light source, both *I*_LO_ and *I*_pr_ changed in proportion to the output power and so we have the relation *I*_LO_^2^ = |*κ*_1_/*κ*_2_|^2^*I*_LO_ × *I*_pr_. Thus the square dependence of the signal that we observed in the figures means that the signal was proportional to *I*_LO_ × *I*_pr_. Combining the two dependences leads to the conclusion that the signal changed in proportion to *I*_LO_ × *I*_pr_ × *P*_p1_ × *P*_p2_, which agreed with the theoretical result provided by Equation (8), and therefore we confirmed that OLCR detected the exact Stokes signal produced by the Brillouin-enhanced four-wave mixing. It is noted that all the solid lines in the figures were plotted by calculating the magnitude *C*_s_*I*_LO_*I*_pr_*P*_p1_*P*_p2_ in units of pA^2^, where *C*_s_= 4.29 × 10^−5^, and the units of (*I*_LO_, *I*_pr_) and (*P*_p1_, *P*_p2_) were restricted to μA and mW, respectively. Since *C*_s_ is the coefficient taken at *δν*_LO×p_ = 2.05 THz and Ω ≈ Ω_B_ in Equation (8), the coefficient should be *C*_s_ʹ = *C*_s_ × (*δν*_LO×p_/*δν*ʹ_LO×p_)^2^ × (Γ_B_Ω_B_)^2^/{(Ω_B_^2^-Ω^2^)^2^ + (Γ_B_Ω)^2^} at arbitrary values of *δν*ʹ_LO×p_ and Ω.

### 4.2. Current Noise Spectral Density

**Figure 5:**
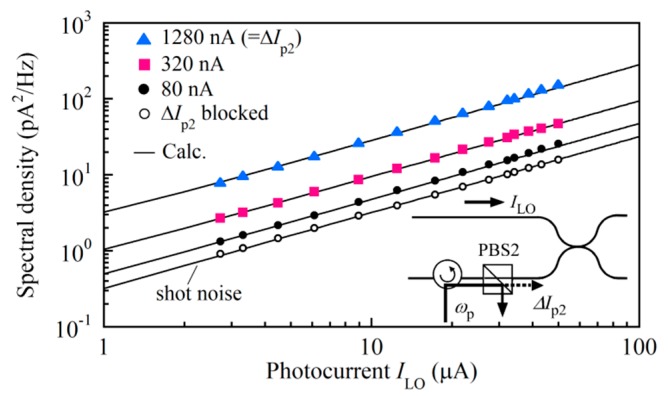
Current noise spectral density as a function of photocurrent *I*_LO_ measured when the power of the pump light at *ω*_p_ that entered the balanced mixer was changed so that the photocurrent was Δ*I*_p2_ = 80, 320 or 1280 nA. Both the probe light and the pump light at *ω*_p_ + Ω were blocked. The photocurrent was Δ*I*_p2_ = 80 nA when the pump light was attenuated by 55 dB. The solid lines were plotted by calculating *σ*^2^ = 2*e*(*I*_LO_ + Δ*I*_p2_) + *σ*^2^_LO×P2_ at different Δ*I*_p2_ values.

The three kinds of light waves entered the balanced mixer to produce beat noise as shown schematically in Figure 3. The current noise spectral density was measured as follows. The vertical scale of the FFT signal analyzer was set at the power spectral density (PSD) in units of V^2^/Hz. We converted the PSD values measured with the analyzer at 5 kHz to the corresponding squared currents by using the gain of the TIA. To show the effect of the beat noise caused by the leaked pump light, we blocked the probe light and the pump light at *ω*_p_ + Ω from entering the DUT by disconnecting the optical path between PBS1 and CL1. The power of the pump light at *ω*_p_ launched into the DUT was 12 mW, and we increased the photocurrent Δ*I*_pr_ of the leaked pump light from 80 to 320 to 1280 nA by finely adjusting its SOP with PC2. We changed the output power of the low coherence light source and measured the current noise spectral density at 5 kHz as a function of the photocurrent *I*_LO_ as shown in Figure 5. It should be noted that the photocurrent of Δ*I*_pr_ = 80 nA was measured when the pump light power was attenuated by 55 dB with PBS2.

At every photocurrent value Δ*I*_P2_, the measured spectral density as a function of *I*_LO_ agreed with the theoretical curve shown by the solid line of *σ*^2^ = 2*e*(*I*_LO_ + Δ*I*_p2_) + *σ*^2^_LO×P2_, which was obtained by letting Δ*I*_pr_ = 0 and Δ*I*_p1_ = 0 in Equation (15). To calculate the values of *σ*^2^_LO×P2_ according to Equation (14), we estimated the effective width defined by Equation (5) at *δν*_LO×p_ = 2.05 THz beforehand from the measured spectrum of the low coherence light output. The shot noise limited spectral density was observed by completely blocking the pump light from entering the DUT as shown by the open circles in the figure, revealing that the inherent intensity noise of the low coherence light was suppressed by the balanced detection. As the pump light power increased, the beat noise overcame the shot noise and the former is shown to be a dominant noise that degrades the DR and SNR of the Brillouin grating measurement.

**Figure 6:**
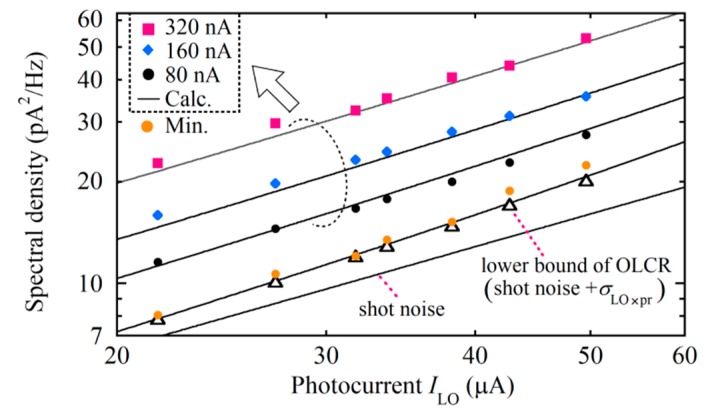
Current noise spectral density as a function of photocurrent *I*_LO_ measured when all the lights were launched and the total power of both pump lights that entered the balanced mixer was changed so that their total photocurrent was Δ*I*_p_ = 80, 160 or 320 nA. The solid lines were plotted by calculating *σ*^2^ = 2*e*(*I*_LO_ + Δ*I*_pr_ + Δ*I*_p_) + *σ*^2^_LO×pr_ + *σ*^2^_LO×p_ at different Δ*I*_p_ values.

Finally, we launched all the lights into the interferometer while the serrodyne modulation to the phase modulators (PM1 and PM3) was switched off to prevent the generation of a Stokes signal at 5 kHz. We measured the spectral density at 5 kHz as a function of *I*_LO_ by increasing the power of the low coherence light output, as shown in Figure 6, where we changed *I*_LO_ within the range of practical interest. The pump light powers *P*_p1_ and *P*_p2_ were 200 and 12 mW, respectively. Since both Δ*I*_p1_ and Δ*I*_p2_ were launched into the mixer, we increased the total photocurrent Δ*I*_p_ = Δ*I*_p1_ + Δ*I*_p2_ from 80 to 160 to 320 nA by making the fine adjustment of the SOP of the pump lights with the PC2. From Equation (15) the total noise was expressed by *σ*^2^ = 2*e*(*I*_LO_ + Δ*I*_pr_ + Δ*I*_p_) + *σ*^2^_LO×pr_ + *σ*^2^_LO×p_, with which we calculated the noise change as shown by the solid lines. Here *σ*^2^_LO×pr_ was evaluated from Equation (11) with the measured reflection of -62 dB and *δν*_LO×pr_ = 2.88 THz, whereas *σ*^2^_LO×p_ was evaluated from 4*I*_LO_Δ*I*_p_/*δν*_LO×p_ with *δν*_LO×p_ = 2.05 THz. The noise *σ*^2^_LO×p_ overcame the lower bound of the shot noise + beat noise *σ*^2^_LO×pr_, which is the main noise of the conventional OLCR system, and so the total noise increased linearly with *I*_LO_. The agreement between the measured and calculated values shows that the noise of the Brillouin-grating-based OLCR is expressed by Equation (15). As long as Δ*I*_p_≥80 nA, we could maintain a given value of Δ*I*_p_ for 20 min under our laboratory conditions. We could temporarily reduce Δ*I*_p_ to a minimal level of the order of 10 nA so that the noise level approached the lower bound, as shown by the open circles in Figure 6.

### 4.3. Relationship between DR and SNR

We fixed the pump light powers *P*_p1_ and *P*_p2_ at 200 and 12 mW, respectively. The output power of the low coherence light was 114 mW so that the photocurrent was *I*_LO_ = 47 μA. The down-conversion frequency was fixed at 10.77 GHz to measure the Stokes signal from the same position at a distance of 2 cm from the joint of the mated fiber connectors. To check the validity of the relationship between the DR and SNR, which we derived as Equation (22), we had to measure the time change of the low-pass filter output under a condition where the bandwidth *VBW* of the low-pass filter was at least twice the *RBW* of the band-pass filter, as described in Appendix A. To meet this condition, we used an analog-type RF spectrum analyzer where we set the center frequency, span, *VBW* and *RBW* at 5 kHz, 0 Hz (or a zero span), 100 Hz, and 30 Hz, respectively. The spectrum analyzer had a video output port from which the square-law detection output was streamed.

**Figure 7:**
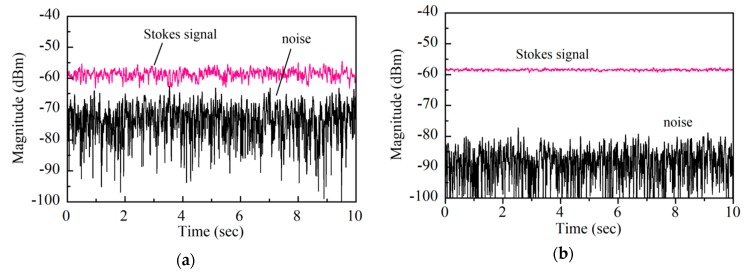
Time change of video output from an RF spectrum analyzer set at 5 kHz, a zero span, *VBW* = 100 Hz and *RBW* = 30 Hz when (**a**) *DR* = 12.5 dB and *SNR* = 3.05 and (**b**) D*R* = 28 dB and *SNR* = 18.1. The linear stage used in the variable delay line was fixed at a particular position and the down-conversion frequency was 10.77 GHz.

We reduced the photocurrent Δ*I*_p_ gradually to reduce the noise, for every value of which we acquired the time changes of the Stokes signal and noise for ten seconds by switching the serrodyne modulation to both phase modulators on and off. Figure 7a shows one example of the acquired waveforms, where the noise (lower trace) was so high that it was close to the Stokes signal (upper trace), which also fluctuated with time. We calculated the standard deviation and mean value of the fluctuating Stokes signal from the acquired waveform, which corresponded to *σ_Y_* in Equation (17), and <*Y*(*t*)> = *A*^2^/2 + *σ*^2^ in Equation (18). 

**Figure 8:**
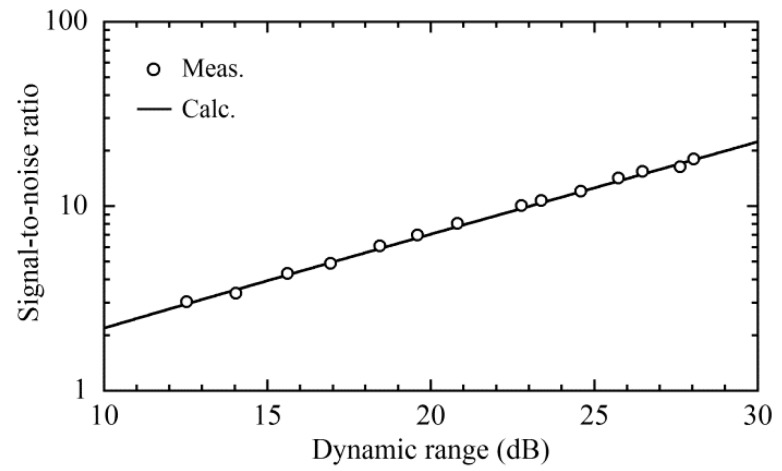
Relationship between dynamic range and signal-to-noise ratio. The dynamic range was changed by changing the power of the pump lights entering the balanced mixer. The solid line is a theoretical curve of the function described by Equation (22).

After calculating the variance *σ*^2^ from the time change of the noise we reduced the photocurrent Δ*I*_p_ gradually to reduce the noise, for every value of which we acquired the time changes of the Stokes signal and noise for ten seconds by switching the serrodyne modulation to both phase modulators on and off. Figure 7a shows one example of the acquired waveforms, where the noise (lower trace) was so high that it was close to the Stokes signal (upper trace), which also fluctuated with time. We calculated the standard deviation and mean value of the fluctuating Stokes signal from the acquired waveform, which corresponded to *σ_Y_* in Equation (17), and <*Y*(*t*)> = *A*^2^/2 + *σ*^2^ in Equation (18). After calculating the variance *σ*^2^ from the time change of the noise waveform, we derived the Stokes signal *A*^2^/2 by substituting *σ*^2^ from the value <*Y*(*t*)>. From this calculation, we estimated the DR from (*A*^2^/2)/*σ*^2^ and the SNR from (*A*^2^/2)/*σ_Y_* of Equation (19) to be 12.5 dB on a logarithmic scale and 3.05 on a linear scale, respectively. As we reduced the photocurrent Δ*I*_p_ by adjusting the SOP of the pump lights which entered the balanced mixer, the fluctuations of both the Stokes signal and the noise decreased to *DR* = 28 dB and *SNR* = 18.1, respectively, as shown in Figure 7b. We estimated both the *DR* and *SNR* values at different photocurrent values of Δ*I*_p_ and plotted their relation in Figure 8 together with the calculation according to Equation (22). The agreement between the measured and calculated values shows that the fluctuations in the Stokes signal originated from the interaction between the Stokes signal and the noise via square-law detection.

### 4.4. Brillouin Grating Reflectogram Revealed with DFS

**Figure 9:**
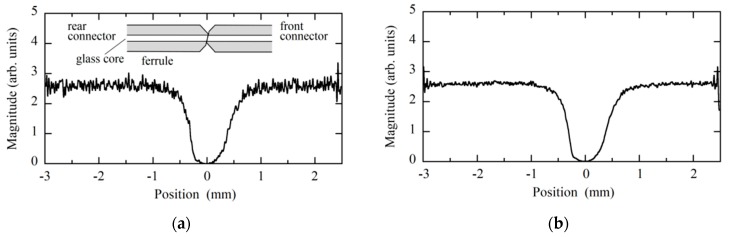
(**a**) Brillouin grating reflectogram around the joint of the mated fiber connectors, which was derived from one scan of the linear stage. (**b**) Mean reflectogram derived by averaging 10 reflectograms. The reflectograms were derived by processing the acquired interferograms with the DFS.

The light powers of the two pump lights and probe light were the same as those in Section 4.3, and the down-conversion frequency was also 10.77 GHz. The photocurrent Δ*I*_p_ of the leaked pump light was adjusted at 80 nA. The reflectogram around the joint of the angle-polished fiber connectors, which we obtained with the DFS, is shown in Figure 9a. We calculated the mean level and standard deviation of the fluctuating signal ranging from −2.5 to −1 cm and estimated the SNR by the ratio of the former to the latter to be 24. The achieved SNR was 6 times higher than that in our previous paper [8]. After performing ten repetitive measurements for 4 min, we had a smoothed reflectogram as shown in Figure 9b, where the SNR was improved to 65, which was the same as that we previously obtained with 200 repetitive measurements.

**Figure 10:**
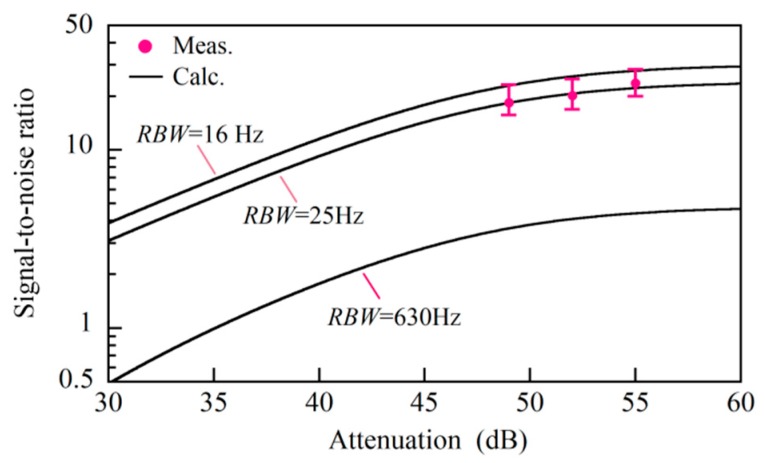
Attenuation dependent signal-to-noise ratio of a Brillouin grating reflectogram that was acquired by processing one interferogram with the DFS. Attenuation was defined by Δ*I*_p_/*I*_p2_ on a logarithmic dB scale, where *I*_p2_ was the photocurrent produced if the pump light at *ω*_p_ were to be detected with a photodiode. Theoretical curves were plotted for three resolution bandwidths of *RBW* = 16, 25 and 630 Hz.

We increased the photocurrent Δ*I*_p_ from 80 to 160 to 320 nA, and at each value we acquired ten reflectograms from the mated fiber connectors and plotted the mean value and distribution of their SNRs along the fiber from -2.5 to -1 cm with the open circles and error bars in Figure 10. The horizontal axis is the attenuation expressed by Δ*I*_p_/*I*_p2_ on a dB scale. While calculating a reflectogram with the DFS, we employed two kinds of rectangular windows for band-pass filtering. We employed a 630-Hz-wide window when we demodulated the signal, and a window ranging from 1527 to 1576 nm in the spectral region. It was not clear a priori which bandwidth should be the resolution bandwidth *RBW* of the square-law detection, which was included in the expression of the DR as <*I*_s_^2^(*t*)>/(*σ*^2^ × *RBW*) = *C*_s_*I*_LO_*I*_pr_*P*_p1_*P*_p2_/(*σ*^2^ × *RBW*), where *C*_s_ = 4.29 × 10^−5^ and *σ*^2^ is denoted by Equation (15). The theoretical SNR at the former bandwidth *RBW* = 630 Hz as a function of the attenuation was too low to fit the measured values, as shown in the figure. By changing the *RBW* until the theoretical curve fitted the measured values, we found that the actual *RBW* was 25 Hz. If the linear stage had translated at a constant speed of 400 μm, the beat frequency produced by the translation would distribute from 508 to 524 Hz depending on the frequency of the individual spectral components. That is, the narrowest bandwidth that we could apply to the interferogram without deformation was *RBW* = 16 Hz, and the resultant SNR is also plotted in the figure. Although the stage did not translate at a constant speed, we concluded that the combination of changing the grid to the equal path increments and the band-pass filtering in the spectral region enabled us to reduce the noise level close to the minimum level.

### 4.5. Examples of OLCR Diagnosis

**Figure 11:**
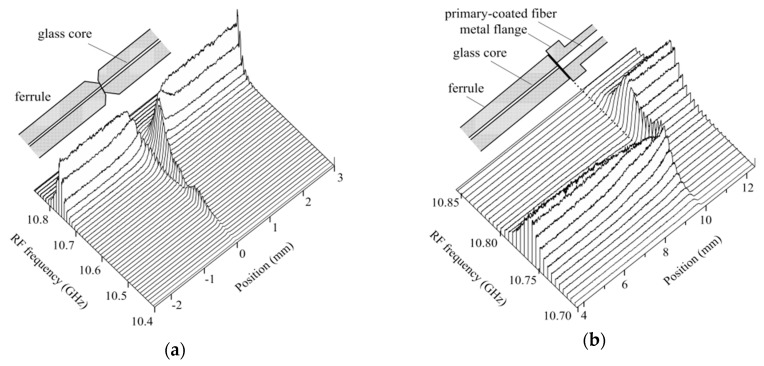
Mean Brillouin grating reflectograms acquired around the (**a**) joint and (**b**) boundary between the ferrule and metal flange of the front connector. The individual reflectograms were derived by processing the interferograms with the DFS.

The light powers of the two pump lights and probe light were the same as those in Section 4.3. Since we succeeded in reducing the measurement time required for obtaining one Brillouin grating reflectogram with a high SNR to 4 min, we were able to obtain the reflectograms from the mated fiber connectors as a function of the down-conversion frequency, which changed from 10.4 to 10.85 GHz in steps of 10 MHz, as shown in Figure 11. Throughout the measurements, every 20 min we made a fine adjustment of PC2 to maintain the leaked pump light at Δ*I*_p_ = 80 nA. Figure 11a shows the reflectograms we obtained around the joint of the glass cores in the rear and front connectors. Since these connectors were spring-loaded and mated, the angle-polished fiber endfaces were pressed together and stress applied to the glass core at a distance of up to 0.5 mm from the joint moved that part of the Brillouin grating to a different frequency region. In the front connector, the part of the fiber from its endface to the base of the glass core, which was 9.8 mm long, passed through a small hole in the ferrule and was fixed to it with adhesive. The rest of the fiber had a primary coating that was fixed to a metal flange with adhesive, as shown in the inset of Figure 11b. The Brillouin grating was generated at 10.76 GHz in the glass core at a distance of 0.5 mm from the joint, moved dynamically in a positive direction along the fiber, suddenly disappeared at the base of the core, which was located at a distance of 9.8 mm, and appeared again at 10.805 GHz to move dynamically through the primary-coated glass core. This propagation means that the primary coating of the fiber fixed in the metal flange relieved the glass core of the stress that was applied in the ferrule by 9.1 × 10^−4^, where we assumed that the strain coefficient was 4.93 MHz/10^−4^ at 1.55 μm [20].

**Figure 12:**
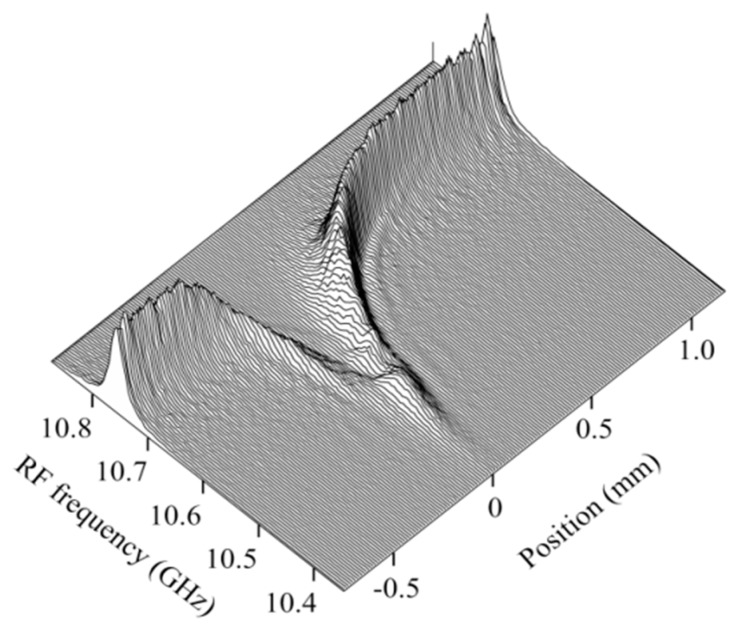
Distribution of Brillouin spectrum around the joint of the mated fiber connectors. The distribution was obtained by first acquiring the Stokes signal as a function of position (or Brillouin grating distribution) at different down-conversion frequencies and then plotting the signal as a function of the frequency at equidistant spaces.

**Figure 13:**
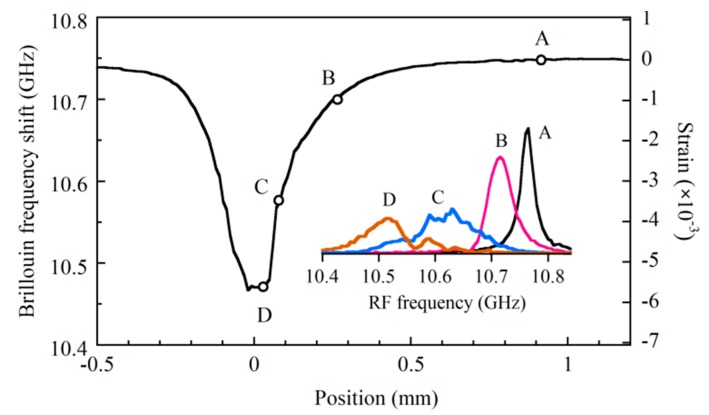
Change in Brillouin frequency shift along the mated fiber connectors. Inset shows Brillouin spectra at particular points A, B, C and D. The frequency shift was rescaled to give the deviation of the strain from that at point A by using a strain constant of 4.93 MHz/10^−4^ at 1.55 μm.

We changed the down-conversion frequency from 10.35 to 10.875 GHz more precisely in steps of 5 MHz and measured the reflectograms around joint of the mated connectors. Then we plotted the Stokes signal as a function of the RF frequency at equidistant spaces along the fiber to display the Brillouin spectrum distribution as shown in Figure 12. The center frequency of each spectrum provided us with the Brillouin frequency shift along the fiber as shown in Figure 13, from which we could observe the change in the strain along the fiber with respect to that at point A by rescaling the vertical axis in the figure. The spectra at the particular positions A, B, C, and D in the front connector are shown by the inset in Figure 13. The spectrum, which we acquired at a certain point with OLCR, is the weighted average of the Brillouin spectra distributed along the fiber within a distance of around 0.1 mm from the point. Therefore, when the stress applied to the glass core increased rapidly within a short distance of 0.5 mm as shown in Figure 13, Brillouin spectra with different frequency shifts contributed to the weighted averaging, resulting in the spectral broadening and deformation shown in the inset.

**Figure 14:**
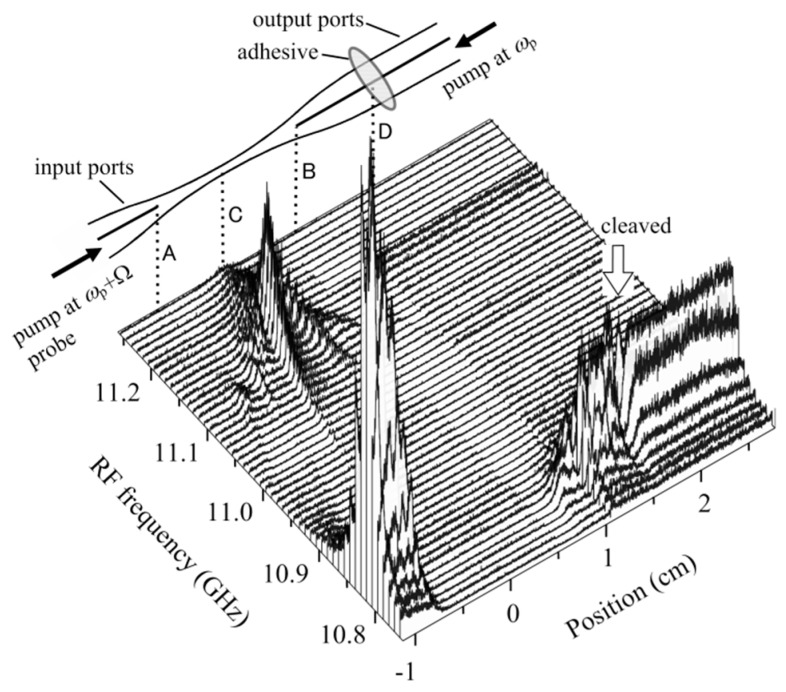
Brillouin grating distributions at different down-conversion frequencies measured around the fused region of a 3-dB fused biconically-tapered single-mode fiber coupler with input and output ports. The coordinates of points A, B, C and D are −8, 8, 0 and 1.5 cm, respectively.

As another example of the OLCR diagnosis, we tested a 3-dB fused biconically tapered single-mode fiber coupler [21] operating at 1.55 μm, as shown in Figure 14. Since the coupler was molded in a package, we could not obtain any information regarding the coupler parameters such as the length of the heated section and the taper size. We assume that the pump light at *ω*_p_ was launched from an output port of the coupler, and that the pump light at *ω*_p_ + Ω and the probe light were launched from an input port. We chose origin C of the position coordinate *z* as the point about which the reflectogram profiles appeared to be symmetrical, and A and B denote the points at *z* = ±0.8 mm. Since outside the line segment AB the Brillouin gratings were generated at the same down-conversion frequency of 10.805 GHz as in the stress-released fiber (as shown in Figure 11b), we considered the outside to consist of the input and output ports of the constituent fibers, whereas the inside was the fused region where the two lowest supermodes were coupling. The reflectograms observed at a 1.5 cm position D were cleaved so sharply that the fiber part was considered to be fixed on an invar plate with adhesive.

The power of the pump light, which was launched from the output port, was attenuated by 3 dB to reach the input port, where the generated Stokes light power should decrease by 3 dB compared with that from the fiber connector. On the other hand, the powers of the pump and probe lights, which were launched from the input port, were both attenuated by 3 dB to reach the output port, where the generated Stokes light power should decrease by 6 dB. After passing through the fused region, the Stokes light power was attenuated by 3 dB to reach the input port, resulting in atotal attenuation of 9 dB. From this estimation, therefore, the Stokes signal observed at the output port was considered to be 6 dB lower than that at the input port, which is consistent with the difference between the signal levels of the input and output ports.

**Figure 15:**
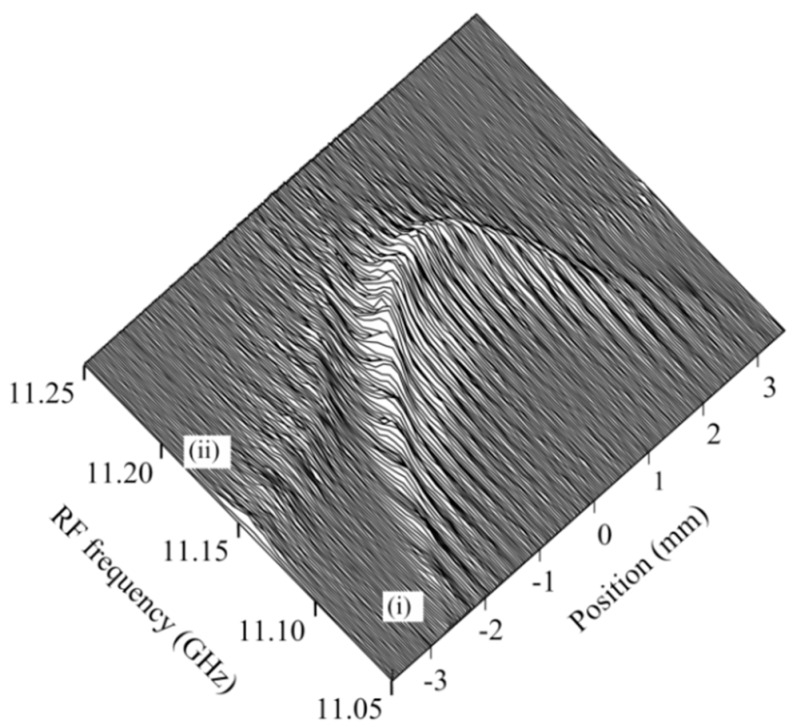
Distribution of Brillouin spectrum around the fused region of the fiber coupler. The main distribution is shown by (ii), which was associated with the small signal distribution shown by (ii).

The reflectograms that we observed in the fused region were so complicated that we performed the reflectogram measurements again by translating the stage over a shorter span of ± 4mm, changing the RF frequency more precisely in steps of 5 MHz, and doubling the number of repetitive measurements to increase the SNR. The resultant Brillouin spectrum distribution around the center is shown in Figure 15. That is, we found that the complicated appearance of the reflectogram distribution arose from the fact that the Brillouin frequency shift changed along the fiber symmetrically with respect to the center from 11.05 to 11.2 GHz, as shown by curve (i). Since the Brillouin frequency shift is closely related to the interaction of the supermodes with the acoustic waves, the frequency shift as a function of the position will provide us with useful information about the longitudinal taper profile, which is an important step when modeling the coupler in the calculation.

Together with the main distribution (i), we observed another small signal distribution shown by (ii) whose Brillouin frequency shift changed gradually from 11.15 to 11.2 GHz. The fused region consisted of the coalesced tapered region and a uniform-waist region. On the assumption that the fused part became a core and the surrounding air was cladding in the latter region, there is a possibility that higher order modes were excited there and propagated along the tapered fiber in the former region.

## 5. Conclusions

We have shown theoretically and experimentally that speckle-like noise was generated during Brillouin grating measurements with micrometer-resolution OLCR by the interaction of the Stokes signal with the beat noise via square-law detection. To reduce the noise we had no choice but to reduce the noise generated by the beat between the LO light and a pump light entering the balanced mixer. This was achieved by using a fiber-optic polarizer and making a fine adjustment to the SOP of the polarizer output. We achieved the SNR of 24 for one Brillouin grating reflectogram and that of 65 by averaging ten individual reflectograms. The achievement of such a high SNR enabled us to acquire a reflectogram as a function of the down-conversion frequency, which provided us with the Brillouin spectrum distributions in mated fiber connectors and a 3-dB fused fiber coupler.

## Appendix A

The autocorrelation function of the output *Y*(*t*) from the square-law device is:

(A1) $$\langle Y(t+\tau )Y(t)\rangle =R_{I\times I}(\tau )+R_{I\times N}(\tau )+R_{N\times N}(\tau ),$$

where:

(A2) $$R_{I\times I}(\tau )=\langle I^{2}(t+\tau )I^{2}(t)\rangle ,$$

(A3) $$R_{I\times N}(\tau )=\langle I^{2}(t+\tau )N^{2}(t)\rangle +\langle I^{2}(t)N^{2}(t+\tau )\rangle +4\langle I(t+\tau )I(t)N(t+\tau )N(t)\rangle ,$$

(A4) $$R_{N\times N}(\tau )=\langle N^{2}(t+\tau )N^{2}(t)\rangle .$$

The first to third terms are generated by the coupling of the signal with itself, that of the signal with the noise, and that of the noise with itself, respectively [19]. Since the Stokes signal is a sinusoidal waveform, we let *I*(*t*) = *A*cos(2*πf*_c_*t* + *θ*). Here we introduce the spectral density function *S_N_*(*f*) of the noise from the band-pass filter that is defined by <*N*(*t* + τ)*N*(*t*)> = ∫_-∞_^+∞^*S_N_*(*f*)exp(−2*πifτ*)*df*. The general form of *S_N_*(*f*) is:

(A5) $$S_{N}(f)=C\sigma^{2}\left\{H(f-f_{c})+H(f+f_{c})\right\},$$

(A6) $$C=1/2\int_{-\infty }^{+\infty }H(f)df,$$

where the function is normalized in such a way that the variance becomes *σ*^2^. We impose the strict condition of *H*(*f*) = 0 at |*f*|>*RBW*/2 on the bandwidth *RBW* of the band-pass filter. By assuming that *N*(*t*) obeys a stationary random Gaussian process, we can calculate these three correlations.

Correspondingly, the spectral density *S_Y_*(*f*), which is the Fourier inverse transform of <*Y*(*t + τ*)*Y*(*t*)>, consists of the constituent spectral densities:

(A7) $$S_{Y}(f)=S_{I\times I}(f)+S_{I\times N}(f)+S_{N\times N}(f),$$

where:

(A8) $$S_{I\times I}(f)=\frac{A^{4}}{4}\delta (f)+\frac{A^{4}}{16}\left\{\delta (f-2f_{c})+\delta (f+2f_{c})\right\}$$

(A9) $$S_{I\times N}(f)=A^{2}\sigma^{2}\delta (f)+A^{2}\left\{S_{N}(f-f_{c})+S_{N}(f+f_{c})\right\})$$

(A10) $$S_{N\times N}(f)=\sigma^{4}\delta (f)+2\int_{-\infty }^{+\infty }S_{N}(f^{\prime })S_{N}(f-f^{\prime })df^{\prime }$$

and <*Y*^2^(*t*)> of the low-pass filter output is:

(A11) $$\langle Y^{2}(t)\rangle =\int_{-\infty }^{+\infty }B(f)\left\{S_{I\times I}(f)+S_{I\times N}(f)+S_{N\times N}(f)\right\}df.$$

We assume in Equation (A11) that the low-pass filter *B*(*f*) has a rectangular window with a height at unity and a bandwidth of *VBW*, as depicted by (*a*) in Figure 3.

Equation (A8) means that the spectral density *S_I_*_×*I*_(*f*) is nonzero only at zero frequency and ±2*f*_c_. By letting the bandwidth be *VBW*<<*f*_c_, the low-pass filter passes the component only at zero frequency, as shown by (*b*) in Figure 3 and so the contribution to <*Y*^2^(*t*)> is:

(A12) $$\int_{-\infty }^{+\infty }B(f)S_{I\times I}(f)df=\frac{A^{4}}{4}.$$

By substituting Equation (A5) into (A9), we have:

(A13) $$S_{I\times N}(f)=A^{2}\sigma^{2}\delta (f)+A^{2}\sigma^{2}C\left\{H(f-2f_{c})+2H(f)+H(f+2f_{c})\right\}.$$

The band-pass filter is so narrow at *RBW*<<*f*_c_ that *H*(*f*), which is centered at zero frequency, and *H*(*f*±2*f*_c_), which is centered at ±2*f*_c_, do not overlap each other. We consider that the low-pass filter blocks the term involving *H*(*f*±2*f*_c_) in Equation (A13) as shown by (*c*) in Figure 3, and the low-pass filter output is:

(A14) $$\int_{-\infty }^{+\infty }B(f)S_{I\times N}(f)df=A^{2}\sigma^{2}+2A^{2}\sigma^{2}C\int_{-\infty }^{+\infty }H(f)df=2A^{2}\sigma^{2}.$$

By substituting Equation (A5) into (A10), we have:

(A15) $$S_{N\times N}(f)=\sigma^{4}\delta (f)+2\sigma^{4}C^{2}\int_{-\infty }^{+\infty }\left\{\begin{array}{l} H(f^{\prime }-f_{c})H(f-f^{\prime }-f_{c})+H(f^{\prime }-f_{c})H(f-f^{\prime }+f_{c})\\ +H(f^{\prime }+f_{c})H(f-f^{\prime }-f_{c})+H(f^{\prime }+f_{c})H(f-f^{\prime }+f_{c}) \end{array}\right\}df^{\prime }.$$

The first term of the integrand in Equation (A15) has a finite value only when |*f*ʹ*-f*_c_|<*RBW*/2 and |*f*-*f*ʹ-*f*_c_|<*RBW*/2, or |*f-*2*f*_c_|<*RBW*, and the contribution to <*Y*^2^(*t*)> from this term becomes negligibly small as a result of the low-pass filtering. In the same way, the fourth term in the integrand can be neglected. As a result, Equation (A15) is simplified to:

(A16) $$S_{N\times N}(f)=\sigma^{4}\delta (f)+4\sigma^{4}C^{2}\int_{-\infty }^{+\infty }H(f^{\prime })H(f-f^{\prime })df^{\prime }$$

whose behavior is depicted by (*d*) in Figure 3. Then the contribution to <*Y*^2^(*t*)> is:

(A17) $$\int_{-\infty }^{+\infty }B(f)S_{N\times N}(f)df=\sigma^{4}+4\sigma^{4}C^{2}{\left\{\int_{-\infty }^{+\infty }H(f)df\right\}}^{2}=2\sigma^{4}.$$

From Equations. (A12), (A14) and (A17), we obtain the final result of <*Y*^2^(*t*)> as:

(A18) $$\int_{-\infty }^{+\infty }B(f)S_{Y}(f)df=\frac{A^{4}}{4}+2A^{2}\sigma^{2}+2\sigma^{4}.$$

Here it is noted that the integrand in the second term on the right-hand side of Equation (A16) is finite only when |*f*ʹ *− f*_c_|<*RBW*/2 and |*f* − *f*ʹ + *f*_c_|<*RBW*/2, and this condition leads to |*f*|<*RBW*. This means that distribution of the second term as a function of *f* is at least twice as wide as that of the band-pass filter. Therefore, when we measure the signal fluctuations, we should use a low-pass filter satisfying the condition *VBW*>2*RBW*.
